# WWC1 deficiency exacerbates sepsis-induced lung injury by promoting NETosis, M1 and M2b macrophage recruitment, and pyroptosis via YAP1 and STING pathways

**DOI:** 10.1186/s12950-026-00488-8

**Published:** 2026-04-17

**Authors:** Yifeng Chen, Han Xu, Chengyang He, Yue Qi, Yaowu Chen

**Affiliations:** 1 Department of Emergency Medicine, People’s Hospital of Lijiang, Lijiang, Yunnan 674100 China; 2 The First Clinical School of Medicine, Shandong University of Chinese Medicine, Jinan, 25000 China; 3 Institute of Pharmacy, Shandong University of Chinese Medicine, Jinan, 25000 China

**Keywords:** WWC1, YAP1, STING, Pyroptosis, NETosis, Macrophage recruitment, Sepsis-induced lung injury

## Abstract

**Background:**

Our recent investigations have shown that WWC1 loss leads to sepsis-induced lung injury (SiLI) by reducing activation of Yes-associated protein 1 (YAP1) and stimulator of interferon genes (STING) pathways. This study further explores the involvements of pyroptosis, NETosis, and macrophage recruitment in the associated events.

**Methods:**

Wild-type (wt) C57BL/6 mice, or mice with WWC1 gene knockout (WWC1-ko) or knock-in (WWC1-ki), were subjected to cecal ligation and puncture for SiLI modeling. Immunofluorescence staining and western blot (WB) were employed to analyze NETosis-related markers in lung tissues. Flow cytometry was employed to analyze the proportions of neutrophils and macrophages in bronchoalveolar lavage fluid (BALF). Immunohistochemistry and WB were applied to analyze pyroptosis markers. Histological staining was performed to analyze inflammatory responses in the lung tissues. Specific antagonists or agonists were utilized to analyze the involvements of YAP1 and STING in the inflammatory cascades.

**Results:**

WWC1-ko mice showed more pronounced NETosis in the lung, accompanied by increased recruitment of neutrophils and macrophages, particularly M1 and M2b subtype macrophages, in the BALF. The pro-pyroptotic cascade was also activated in the lung of WWC1-ko mice. Administration of specific antagonists of NLRP3, YAP1, STING, and IRF3 reduced pyroptosis, alleviated NETosis, reduced abundance of macrophages, and mitigated inflammatory damage in the lung of WWC1-ko mice. By contrast, WWC1-ki mice were more resistant to these inflammatory cascades, which were, however, diminished upon the artificial activation of YAP1 or STING.

**Conclusion:**

The activation of pyroptosis, YAP1, and STING cascades upon WWC1 deficiency contributes to NETosis and accumulation of pro-inflammatory immune cells in SiLI.

**Supplementary Information:**

The online version contains supplementary material available at 10.1186/s12950-026-00488-8.

## Introduction

Acute lung injury (ALI) is a critical respiratory condition marked by widespread pulmonary edema resulting from damage to the alveoli. Clinically, it presents as persistent hypoxemia and respiratory distress [1]. The underlying causes of ALI are diverse, ranging from direct harm to the lungs to indirect damage induced by excessive inflammation ([2, 3]). Sepsis stands out as the primary risk factor, with the lungs being particularly vulnerable to the intense immune response triggered during sepsis, especially in the stage of multi-organ dysfunction [4–6]. Nearly half of sepsis patients develop ALI [7], and this condition, along with its more severe manifestation, acute respiratory distress syndrome (ARDS), remains one of the primary contributors to mortality in septic patients [8].

Sepsis-induced lung injury (SiLI) is primarily defined by an exaggerated inflammatory reaction, damage to the alveolar-capillary barrier, the accumulation of fluid in the lungs, and resulting respiratory dysfunction. The progression of SiLI involves an array of immune and non-immune cells [9]. Neutrophil plays a central role in the pathology of ALI. Their excessive activation and accumulation in the lung tissue is a hallmark of the disease [10–13]. While these neutrophils assist in pathogen clearance, they also contribute to collateral damage to lung tissue by releasing cytotoxic substances, including proteases, oxidants, proteases, and neutrophil extracellular traps (NETs) [5, 11, 14]. NETs are formed through a specialized type of cell death in neutrophils known as NETosis [15, 16]. These extracellular structures consist of chromatin and proteins like myeloperoxidase (MPO), citrullinated histone H3 (CiH3), and neutrophil elastase [16]. The excessive formation of NETs has been implicated in amplifying inflammation and worsening lung injury [17, 18].

Macrophages, which make up about 55% of the immune cells in the lungs [19], are another critical cell type in SiLI. These highly adaptable cells can assume distinct functional states based on the signals they receive from their microenvironment [20]. Macrophages are generally classified into two main types: classically activated (M1) macrophages, which drive inflammatory responses, and alternatively activated (M2) macrophages, which promote tissue repair and have anti-inflammatory properties [21]. These cells exert critical functions in both ALI development and the subsequent tissue repair processes [22].

Recent studies from our team have revealed that WWC1, a crucial regulator in the Hippo signaling, plays a protective role in mitigating inflammation and tissue damage in mice with SiLI. This protective effect is primarily achieved through the promotion of phosphorylation of large tumor suppressor proteins 1 (LATS1) and Yes-associated protein 1 (YAP1), as well as by suppressing the cyclic GMP-AMP synthase (cGAS)-stimulator of interferon genes (STING) pathway simultaneously. Additionally, we discovered that interferon regulatory factor 3 (IRF3), a key effector of the STING pathway, binds to the WWC1 promoter to suppress its transcription, thereby creating a negative feedback loop (data not yet published). Despite these findings, the roles of these molecules in processes such as NETosis and the polarization and recruitment of macrophages during SiLI remain poorly understood. Furthermore, both the YAP1 [23] and STING [24] pathways have been implicated in promoting pyroptosis-a type of programmed cell death triggered by inflammatory signals and mediated by Gasdermin D (GSDMD) proteins [25]. Pyroptosis has also been closely associated with the progression of SiLI [26]. In light of these observations, this investigation aims to further explore the functions of WWC1 and its related molecules in modulating pyroptosis and immune cell recruitment within the context of SiLI.

## Materials and methods

### Animal modeling

Wild-type (wt) C57BL/6 mice, and mice with WWC1 gene knockout (WWC1-ko) or knock-in (WWC1-ki) (aged 8–10 weeks), were provided by Anburui Technology Co., Ltd. (Fuzhou, Fujian, China). All mice were kept under standard experimental conditions with *ad libitum* access to rodent chow and drinking water.

SiLI in mice was generated by cecal ligation and puncture (CLP). The CLP procedure involved anesthesia, ligation of the cecum using 4–0 silk thread, followed by a single puncture using an 18-gauge needle. After expressing a small amount of feces, the cecum was repositioned, and the abdominal wall was sutured. Twenty-four hours after the CLP procedure, mice were euthanized by excessive anesthesia (150 mg/kg nembutal, i.p.), and lung tissues, bronchoalveolar lavage fluid (BALF), and serum samples were harvested for subsequent use.

### Drug treatment

During the experiment, WWC1-ko mice received the following drug interventions: NETosis antagonist **MPO-IN-8** (IN8, 10 mg/kg), M1 macrophage selective antagonist **Momordicoside G** (MDG, 20 mg/kg), NLR family pyrin domain containing 3 (NLRP3) inflammasome antagonist D359-0396 (30 mg/kg), YAP1 antagonist CA3 (50 mg/kg), STING antagonist C-170 (30 mg/kg), or IRF3 antagonist BAY985 (10 mg/kg). WWC1-ki mice were given the LATS1/2 antagonist TRULI (20 mg/kg) or the STING agonist **Vadimezan** (VDZ, 50 mg/kg), Drugs were administered via intraperitoneal injection, with the solvent being dimethyl sulphoxide or phosphate-buffered saline (PBS), as appropriate. All drug interventions were administered via intraperitoneal injection 1 hour after the completion of the CLP surgery. For control treatment, mice were given an equivalent amount of corn oil. Experimental design and timeline is displayed in Fig S4.

### Hematoxylin and eosin (HE) staining

Lung tissues were fixed for 24 h, followed by routine paraffin embedding to prepare 4 μm thick sections. Following deparaffinization and hydration, the sections underwent hematoxylin for 5 min and eosin staining for 3 min. The stained sections were subsequently dehydrated, cleared, and mounted for microscopic analysis. The tissue damage was assessed according to the pathology scoring system.

### Periodic acid-schiff (PAS) staining

After deparaffinization, the sections were placed in 0.5% periodic acid solution for oxidation. After washing with distilled water, Schiff’s reagent was added to stain for 15 min, followed by rinsing with water for color development. Afterward, the sections were counterstained, cleared, and mounted for microscopic analysis. The distribution and intensity of inflammatory regions were recorded.

### Masson’s trichrome staining

Paraffin sections were deparaffinized, and staining was performed following the Masson’s trichrome staining kit protocol (Solarbio Science & Technology Co., Ltd., Beijing, China). The process involved staining with Weigert’s iron hematoxylin, followed by treatment with acid fuchsin and differentiation using phosphomolybdic acid. Collagen fibers were stained with aniline blue for 5 min. The sections were cleared and mounted for microscopic analysis. The fibrotic area was quantified.

### Immunofluorescence staining

The lung tissue sections were processed with cryosectioning to prepare 6 μm thick frozen sections. After fixation, the sections were washed, then permeabilized with 0.1% Triton X-100 for 10 min. Following blocking with 5% BSA, the sections were incubated overnight (4°C) with antibodies against MPO (1: 200), CiH3 (1: 200), and peptidyl arginine deiminase 4 (PAD4) (1: 200 dilution). The following day, the sections were incubated with Alexa Fluor-conjugated IgG (1: 500) for 1 h. Nuclei were stained with DAPI. Images were captured using a fluorescence microscope.

### Immunohistochemistry (IHC)

After deparaffinization, prepared tissue sections underwent antigen retrieval with citrate buffer (pH 6.0) at high temperature for 15 min. After cooling, endogenous peroxidase activity was inhibited with 3% H_2_O_2_. The sections were then blocked with 5% BSA for 1 h. Afterward, the sections underwent overnight incubation (4°C) with antibodies against NLRP3 (1:200 dilution). The next day, the sections were washed with PBS and probed with HRP-conjugated secondary antibody for 1 h. After DAB staining, sections were counterstained and prepared for microscopic analysis. Images were captured under the microscope and staining intensity was analyzed.

### Western blot (WB) analysis

Total protein from lung tissue was isolated using RIPA lysis buffer and quantified by the BCA method (Thermo Fisher Scientific, Rockford, IL, USA). Equal amounts (20 μg) of proteins were subjected to SDS-PAGE for separation. The proteins were loaded to a PVDF membrane and blocked with 5% non-fat milk for 2 h. The membrane was probed with antibodies (anti-PAD4, anti-CiH3, anti-NLRP3, anti-pro-caspase-1, anti-cleaved-caspase-1, and anti-GSDMD-N, 1:1000) overnight. The next day, the membrane was washed with PBS and probed with secondary antibodies. Blot signals were developed using an ECL kit and quantified utilizing Image J.

### Measurement of cell-free dsDNA and CitH3

To quantify the levels of cell-free double-stranded DNA (dsDNA) in the BALF and serum, the Quant-iT PicoGreen dsDNA assay kit (Invitrogen, Carlsbad, CA, USA) was employed following the manufacturer’s instructions. Briefly, BALF and serum samples were diluted in TE buffer and incubated with the PicoGreen reagent for 5 min at room temperature in the dark. Fluorescence intensity was measured using a microplate reader at an excitation wavelength of 480 nm and an emission wavelength of 520 nm. The concentration of dsDNA was calculated based on a standard curve generated using lambda DNA standards. For the quantitative detection of Citrullinated Histone H3 (CitH3), a specific ELISA kit (Clone Cloud-Clone Corp, Wuhan, China) was utilized. BALF and serum samples were added to the pre-coated microplates and incubated for 1 hour at 37 °C. After washing, biotin-conjugated antibodies and Horseradish Peroxidase (HRP)-avidin were added sequentially. The optical density (OD) was measured at 450 nm using a microplate reader, and the concentration of CitH3 was determined by comparing the OD values to the standard curve.

### Flow cytometry

BALF single-cell suspensions were prepared and Fc-blocked to prevent non-specific binding. Cells were then stained with fluorochrome-conjugated antibodies for 30 minutes at 4 °C in the dark. The gating strategy was as follows: first, cellular debris and dead cells were excluded based on Forward Scatter (FSC) and Side Scatter (SSC) properties. Doublets were excluded using FSC-Area vs. FSC-Height. Neutrophils were identified as CD11b^+^ Ly6G^+^ events. Macrophages were identified as F4/80+ CD11b+ events. From the macrophage population, M1 subtypes were gated as iNOS^+^, and M2 subtypes were analyzed based on CD163 and CD206 expression. Specifically, the M2b subtype was identified by gating for F4/80^+^ CD86^+^ IL-10^+^ cells. Data acquisition was performed on a BD FACSCanto II flow cytometer, and analysis was conducted using FlowJo software.

### Statistical analysis

All data are expressed as mean ± standard deviation, and statistical analysis was conducted utilizing Prism 9.0 software (GraphPad, La Jolla, CA, USA). Independent sample *t*-tests were employed for comparisons between two groups, while one-way analysis of variance followed by Tukey’s post-hoc examinations was employed for comparisons across multiple groups. The significance level was set at *p* < 0.05, and significant differences were marked with *p*-values in statistical plots.

## Results

### WWC1 knockout aggravates SiLI via NETosis and recruitment of M1 and M2b macrophages

**Fig. 1:**
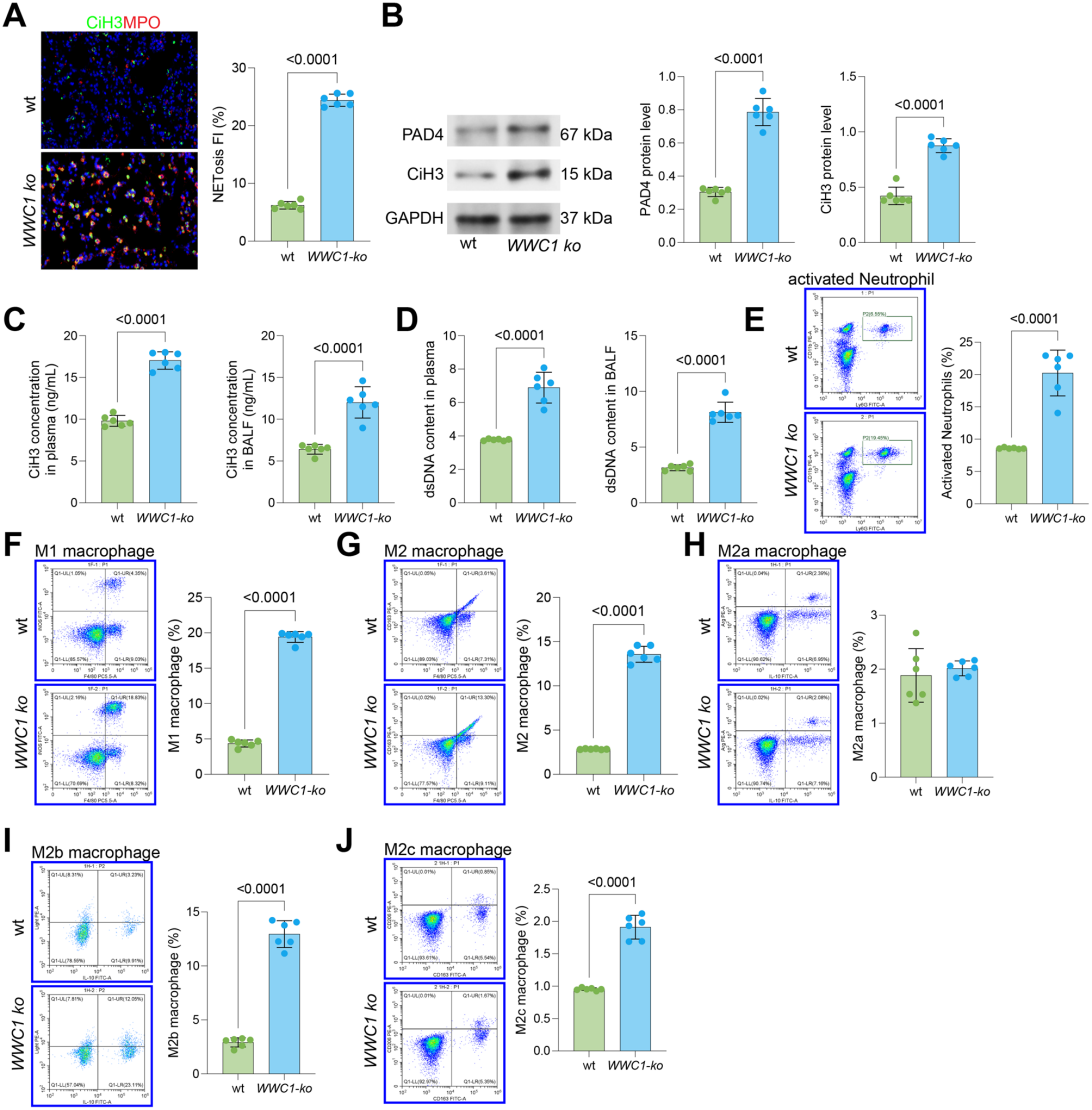
WWC1 knockout aggravates SiLI via NETosis and recruitment of M1 and M2b macrophages. SiLI was induced in wt or WWC1-ko mice via CLP. **A**, Immunofluorescence analysis of MPO and CiH3 fluorescence intensity in the lung tissue of mice; **B**, WB analysis of PAD4 and CiH3 protein expression in the lung tissue of mice; **C**, ELISA analysis of CiH3 levels in the BALF and serum of mice; **D**, PicoGreen assay measuring dsDNA levels in the BALF and serum of mice; **E**, Flow cytometric analysis of activated neutrophils (CD11b^+^Ly6G^+^) in the BALF of mice; **F-G**, Flow cytometric analysis of M1 macrophages (F4/80^+^iNOS^+^) and M2 macrophages (F4/80^+^CD163^+^) in the BALF of mice; **H-J**, Flow cytometric analysis of M2a (IL10^+^Arg1^+^), M2b (CD86^+^IL10^+^Light^+^), and M2c (CD163^+^CD206^+^) in the BALF of mice. Each group contained 6 mice. *p*-values are indicated in the statistical graphs, with *p* < 0.05 considered significant

SiLI was induced in wt and WWC1-ko mice through CLP. A substantial increase in the number of neutrophils and macrophages was detected in the BALF of the WWC1-ko mice versus the wt mice [Fig. S1A–B]. Additionally, research has highlighted a marked increase in NETosis across various lung injury contexts [27, 28]. To examine NETosis in the context of SiLI, immunofluorescence staining was performed to examine NETosis markers, MPO and CiH3, in the mouse lung tissue. The staining intensities of these markers were significantly enhanced in the lung of WWC1-ko mice [Fig. 1A]. Supporting this, WB analysis demonstrated that the protein levels of PAD4, a major regulator in NETosis by catalyzing the citrullination of histones, and CiH3, were prominently elevated in the lung tissue of WWC1-ko mice [Fig. 1B].

Since NETs’ double-strand DNA (dsDNA) and CiH3 can be released into the serum and other bodily fluids, we measured their levels in both mouse serum and BALF. As expected, the dsDNA and CiH3 levels were markedly higher in the WWC1-ko mice than the wt mice [Fig. 1C–D]. Moreover, flow cytometry confirmed a significant increase in activated neutrophils (CD11b^+^Ly6G^+^) in the BALF of the WWC1-ko mice [Fig. 1E].

Given the substantial increase in macrophages and the well-established link between macrophage phenotype and inflammatory responses, we investigated macrophage polarization in these mice. Notably, a pronounced accumulation was observed in M1 macrophages (F4/80^+^iNOS^+^) in the BALF of the WWC1-ko mice [Fig. 1F]. Meanwhile, a notable increase in M2 macrophages (F4/80^+^CD163^+^) was observed in this condition [Fig. 1G]. This result was quite surprising, as M2 macrophages are typically considered anti-inflammatory. However, recent studies suggest that different M2 subtypes, like M2a, M2b, and M2c can have distinct roles [29]. We found that the population of M2b macrophages (CD86^+^IL10^+^Light^+^) was substantially increased in the WWC1-ko mice, while the numbers of M2a (IL10^+^Arg1^+^) and M2c (CD163^+^CD206^+^) macrophages were not significantly reduced [Fig. 1H–J]. These findings suggest that WWC1 loss exacerbates SiLI by promoting NETosis and enhancing the infiltration of M1 and M2b macrophages. To further characterize the inflammatory microenvironment driving this immune cell recruitment, we analyzed the levels of key chemokines and cytokines in the BALF. Consistent with the enhanced infiltration of neutrophils and macrophages, WWC1-ko mice exhibited significantly elevated levels of the neutrophil chemoattractants CXCL1 and CXCL2, as well as the monocyte chemoattractant CCL2, compared to wt mice (Fig. S2A–C). Furthermore, the levels of major pro-inflammatory cytokines, including TNF-α, IL-1β, and IL-6, were also markedly upregulated in the WWC1-deficient group (Fig. S2D–F). These data suggest that the loss of WWC1 exacerbates the inflammatory milieu, thereby promoting the recruitment and activation of innate immune cells.

### Treatment with IN8 or MDG alleviates lung injury in WWC1-ko mice

**Fig. 2:**
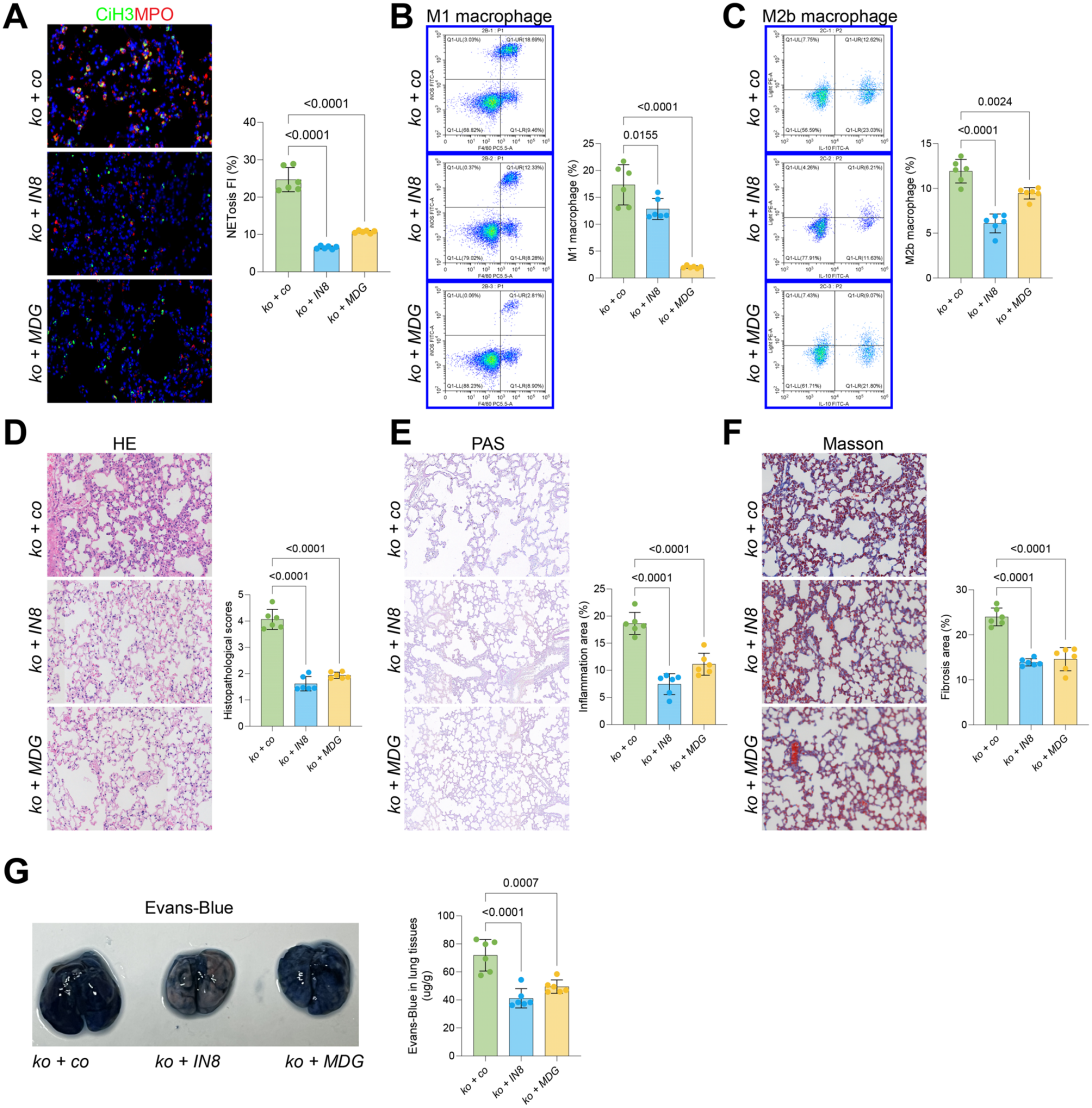
Treatment with IN8 or MDG alleviates lung injury in WWC1-ko mice. WWC1-ko mice were administered corn oil, an active MPO inhibitor IN8, or an M1 macrophage selective inhibitor MDG. **A**, Immunofluorescence analysis of MPO and CiH3 fluorescence intensity in the lung tissue of mice; **B-C**, Flow cytometric analysis of M1 (F4/80^+^iNOS^+^) and M2b (CD86^+^IL10^+^Light^+^) macrophages in the BALF of mice; **D**, HE staining to assess pathological damage in the lung tissue of mice; **E**, PAS staining to evaluate inflammation intensity in the lung tissue of mice; **F**, Masson’s trichrome staining to assess fibrosis in the lung tissue of mice; **G**, Evans blue staining to detect lung tissue permeability in mice. Each group contained 6 mice. Statistical differences between groups were analyzed using ANOVA. *p*-values are indicated in the statistical graphs, with *p* < 0.05 considered significant. Each group contained 6 mice. *p*-values are indicated in the statistical graphs, with *p* < 0.05 considered significant

To further confirm the involvements of NETosis and macrophages in SiLI aggravation upon WWC1 knockout, these WWC1-ko mice were administered an active MPO inhibitor IN8 or an M1 macrophage selective inhibitor MDG. Importantly, the treatment with either IN8 or MDG effectively attenuated NETosis markers in the lung [Fig. 2A], accompanied by decreased proportions of M1 and M2b macrophages in the BALF [Fig. 2B–C]. These treatments also led to a substantial alleviation in lung injury [Fig. 2D] and a reduction in areas of inflammation and fibrosis [Fig. 2E–F]. The assessment of lung permeability revealed a substantial decrease in Evans blue dye content after IN8 or MDG treatment [Fig. 2G]. This evidence suggests that NETosis and macrophage infiltration contribute to lung permeability and inflammatory damage within tissues.

### WWC1 knockout exacerbates pyroptosis in the lung of septic mice

**Fig. 3:**
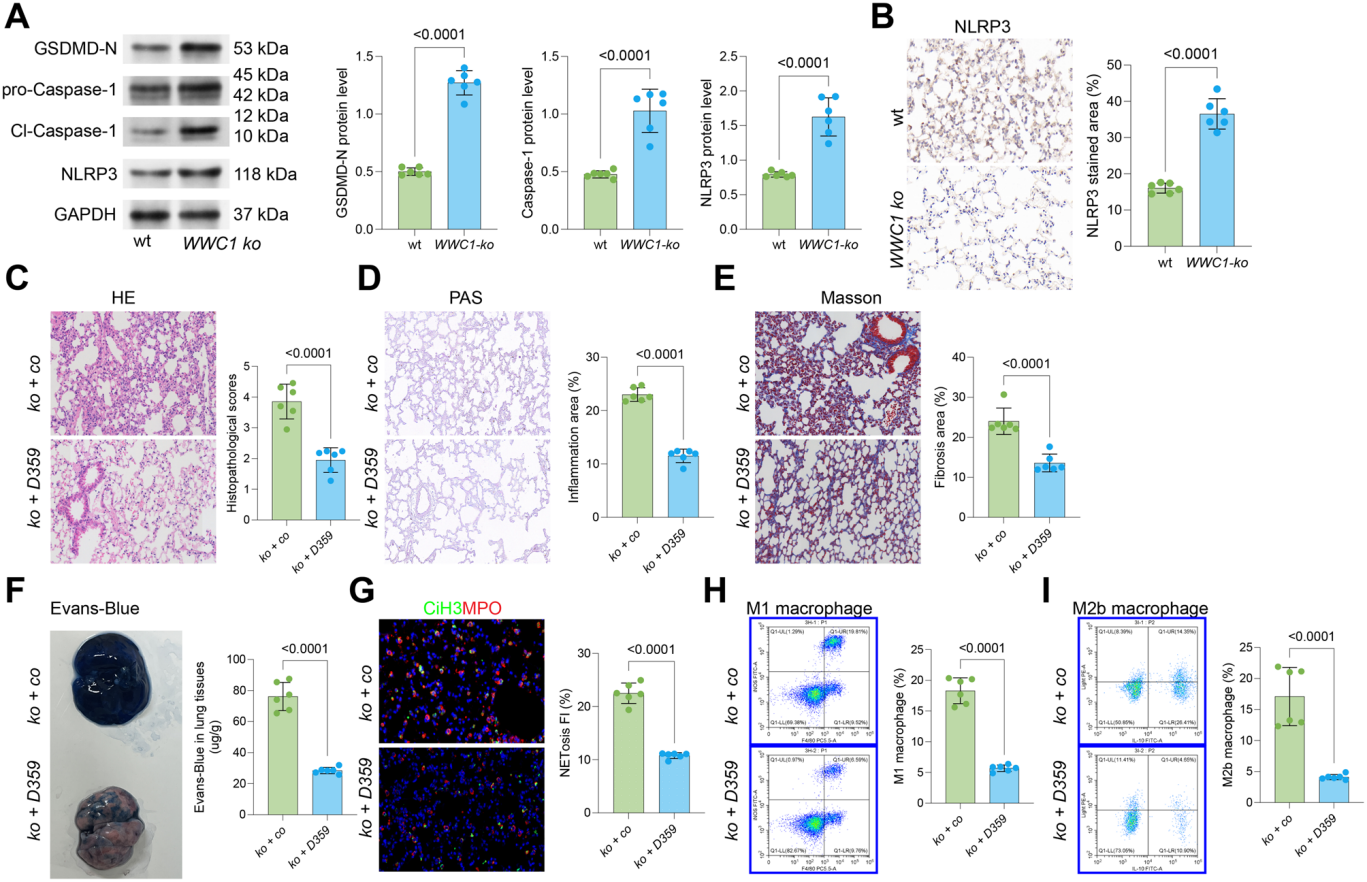
WWC1 knockout exacerbates pyroptosis in the lung of septic mice. **A**, WB analysis to detect protein levels of GSDMD-N, cleaved-caspase-1, and NLRP3 in lung tissue of wt and WWC-ko mice; **B**, IHC analysis of NLRP3 expression in lung tissue of wt and WWC-ko mice. WWC1-ko mice were treated with corn oil or the NLRP3 inflammasome-specific inhibitor D359-0396. **C**, HE staining to assess pathological damage in the lung tissue of mice; **D**, PAS staining to evaluate inflammation intensity in the lung tissue of mice; **E**, Masson’s trichrome staining to assess fibrosis in the lung tissue of mice; **F**, Evans blue staining to detect lung tissue permeability in mice; **G**, immunofluorescence analysis of MPO and CiH3 fluorescence intensity in the lung tissue of mice; **H-I**, flow cytometric analysis of M1 (F4/80^+^iNOS^+^) and M2b (CD86^+^IL10^+^Light^+^) macrophages in the BALF of mice. Each group contained 6 mice. *p*-values are indicated in the statistical graphs, with *p* < 0.05 considered significant

Our recent investigations have revealed that WWC1 loss leads to reductions in LATS1 phosphorylation, which in turn decreases LATS1-mediated phosphorylation of YAP1. Interestingly, YAP1 has been associated with reduced NLRP3-mediated pyroptosis in myocardial injury [23]. Given these insights, we hypothesized that pyroptosis might also contribute to the progression of SiLI in WWC1-ko mice. Indeed, WB analysis suggested that the levels of pyroptosis-related markers, including GSDMD-N, cleaved-caspase-1, and NLRP3, were substantially upregulated in the lung tissue of WWC1-ko mice [Fig. 3A]. Supporting this evidence, IHC assay further confirmed elevated NLRP3 expression in the lung of WWC1-ko mice [Fig. 3B].

To probe into the function of pyroptosis in SiLI, WWC1-ko mice were administered the NLRP3 inflammasome-specific inhibitor D359-0396. This treatment caused a significant reduction of pathological damage in the lung tissue, including alleviated inflammation, reduced mucus production, and decreased collagen deposition [Fig. 3C–E], as well as a notable improvement in lung tissue permeability [Fig. 3F].

Furthermore, the effects of D359-0396 treatment on NETosis and macrophage infiltration were explored. After D359-0396 treatment, the levels of MPO and CiH3 in lung tissue were significantly decreased [Fig. 3G], and the infiltration of both M1 and M2b macrophages was notably reduced [Fig. 3H–I].

### YAP1 antagonist reduces NETosis, macrophage infiltration, and pyroptosis in septic mice

**Fig. 4:**
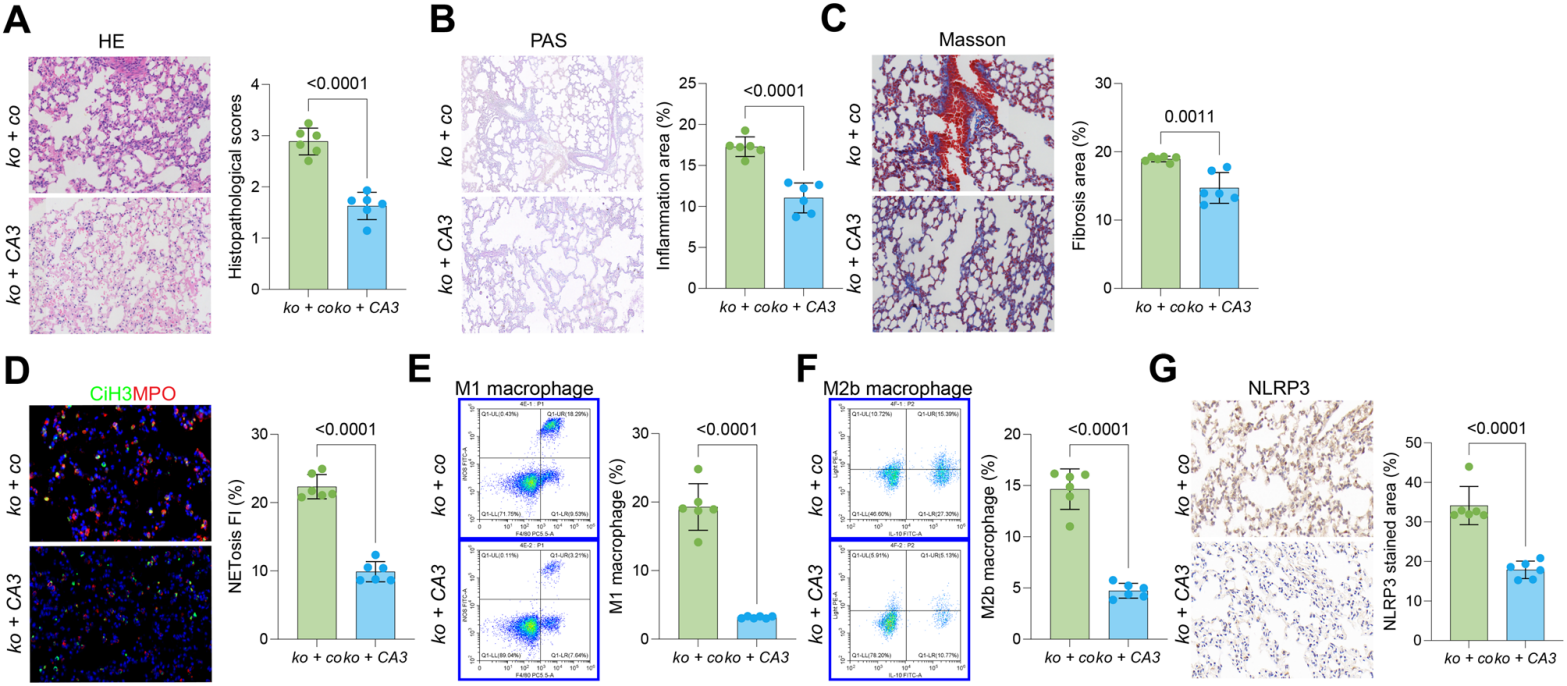
YAP1 inhibition reduces NETosis, macrophage infiltration, and pyroptosis in septic mice. WWC1-ko mice were administered corn oil or the YAP1 antagonist CA3. **A**, HE staining to assess pathological damage in the lung tissue of mice; **B**, PAS staining to evaluate inflammation intensity in the lung tissue of mice; **C**, Masson’s trichrome staining to assess fibrosis in the lung tissue of mice; **D**, Immunofluorescence analysis of MPO and CiH3 fluorescence intensity in the lung tissue of mice; **E-F**, flow cytometric analysis of M1 (F4/80^+^iNOS^+^) and M2b (CD86^+^IL10^+^Light^+^) macrophages in the BALF of mice; **G**, IHC analysis of NLRP3 expression in lung tissue of mice. Each group contained 6 mice. *p*-values are indicated in the statistical graphs, with *p* < 0.05 considered significant

Next, WWC1-ko mice were administered the YAP1 antagonist CA3 (50 mg/kg) to investigate whether WWC1 knockout influences NETosis, macrophage infiltration, and pyroptosis through YAP1 activation. The CA3 treatment significantly mitigated SiLI in WWC1-ko mice, including substantial reductions in tissue damage, inflammation, and fibrosis [Fig. 4A–C].

The CA3 treatment also markedly reduced NETosis in the lung tissue of WWC1 knockout mice [Fig. 4D]. Additionally, the populations of both M1 and M2b macrophages in the BALF were significantly reduced upon CA3-mediated YAP1 inhibition [Fig. 4E–F]. Moreover, the staining intensity of NLRP3 in the lung of CA3-treated mice was also markedly decreased [Fig. 4G].

### STING or IRF3 inhibition alleviates lung injury in WWC1-ko mice by reducing NETosis and pyroptosis

**Fig. 5:**
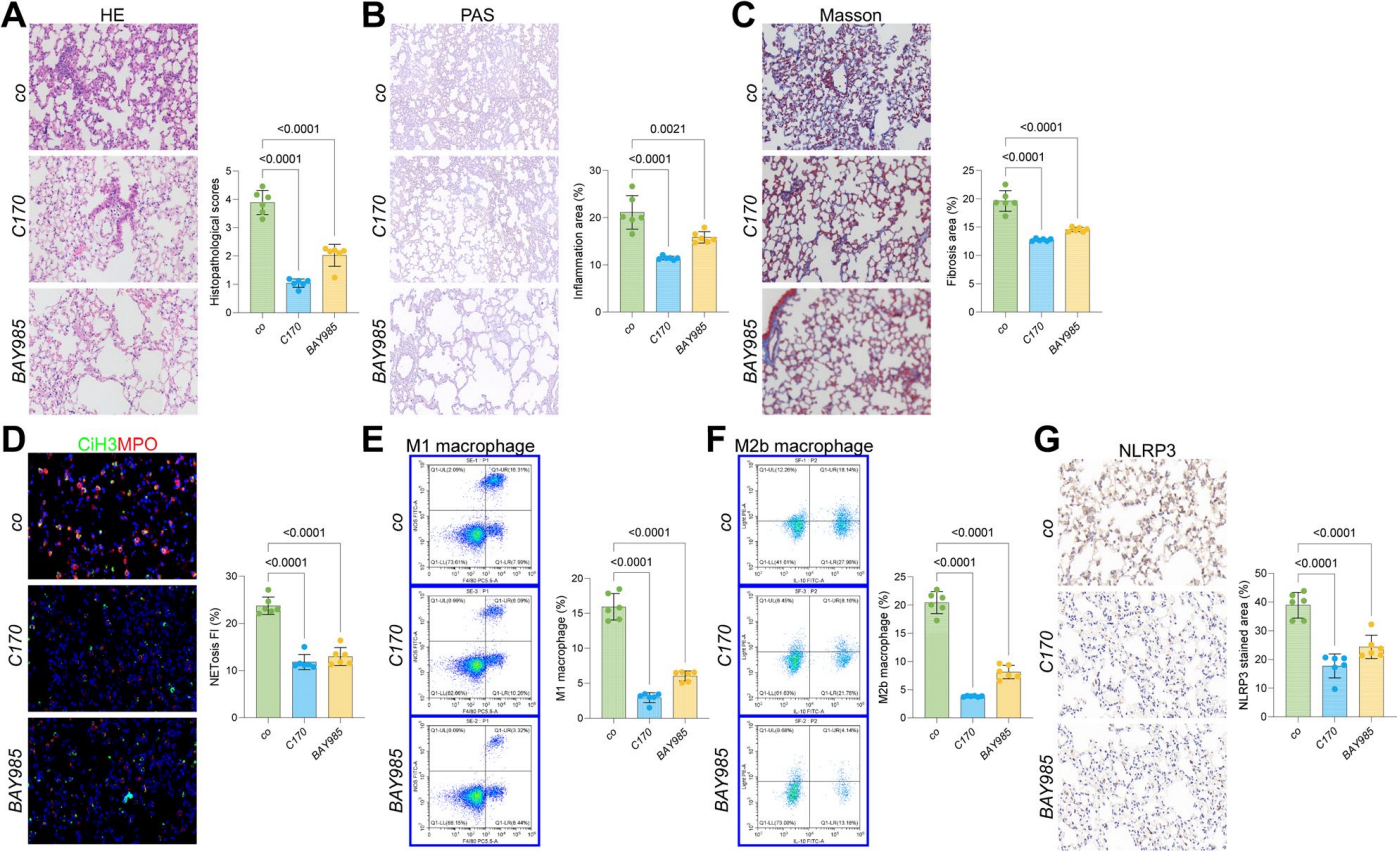
STING or IRF3 inhibition alleviates lung injury in WWC1-ko mice by reducing NETosis and pyroptosis. WWC1-ko mice were administered corn oil, the STING antagonist CA3, C-170, or the IRF3 antagonist BAY985. **A**, HE staining to assess pathological damage in the lung tissue of mice; **B**, PAS staining to evaluate inflammation intensity in the lung tissue of mice; **C**, Masson’s trichrome staining to assess fibrosis in the lung tissue of mice; **D**, immunofluorescence analysis of MPO and CiH3 fluorescence intensity in the lung tissue of mice; **E-F**, Flow cytometric analysis of M1 (F4/80^+^iNOS^+^) and M2b (CD86^+^IL10^+^Light^+^) macrophages in the BALF of mice; **G**, IHC analysis of NLRP3 expression in lung tissue of mice. Each group contained 6 mice. *p*-values are indicated in the statistical graphs, with *p* < 0.05 considered significant

In the previous study, we identified a WWC1-STING-IRF3 feedback loop that plays a pivotal role in SiLI, where WWC1 suppresses the activity of the cGAS-STING pathway and subsequent IRF3 expression, while IRF3 represses WWC1 transcription (data not published yet). Based on these findings, we treated WWC1-ko mice with the STING antagonist C-170 or the IRF3 antagonists BAY985. Following treatment with either C-170 or BAY985, we observed significant alleviation of lung injury symptoms in WWC1-ko mice [Fig. 5A–C].

When it comes to the role of STING-IRF3 cascade in NETosis and macrophage infiltration, found that both C-170 and BAY985 treatments reduced the immunofluorescence staining intensities of CiH3 and MPO in the lung tissue of WWC1-ko mice [Fig. 5D]. Meanwhile, the populations of M1 and M2b macrophages in the BALF were significantly reduced after C-170 or BAY985 treatment [Fig. 5E–F]. Additionally, IHC assay demonstrated that the NLRP3 staining intensity in the lung tissue of C-170 or BAY985-treated WWC1-ko mice was markedly reduced as well [Fig. 5G].

### WWC1 regulates inflammatory cell infiltration and pyroptosis via YAP1 and STING pathways

To further investigate the functions of WWC1, LATS/YAP1, and the STING pathways in SiLI, we further induced SiLI in WWC1 knock-in (ki) mice through CLP, followed by treatments of the LATS1/2-specific inhibitor TRULI to activate YAP1, or the STING agonist VDZ.

**Fig. 6:**
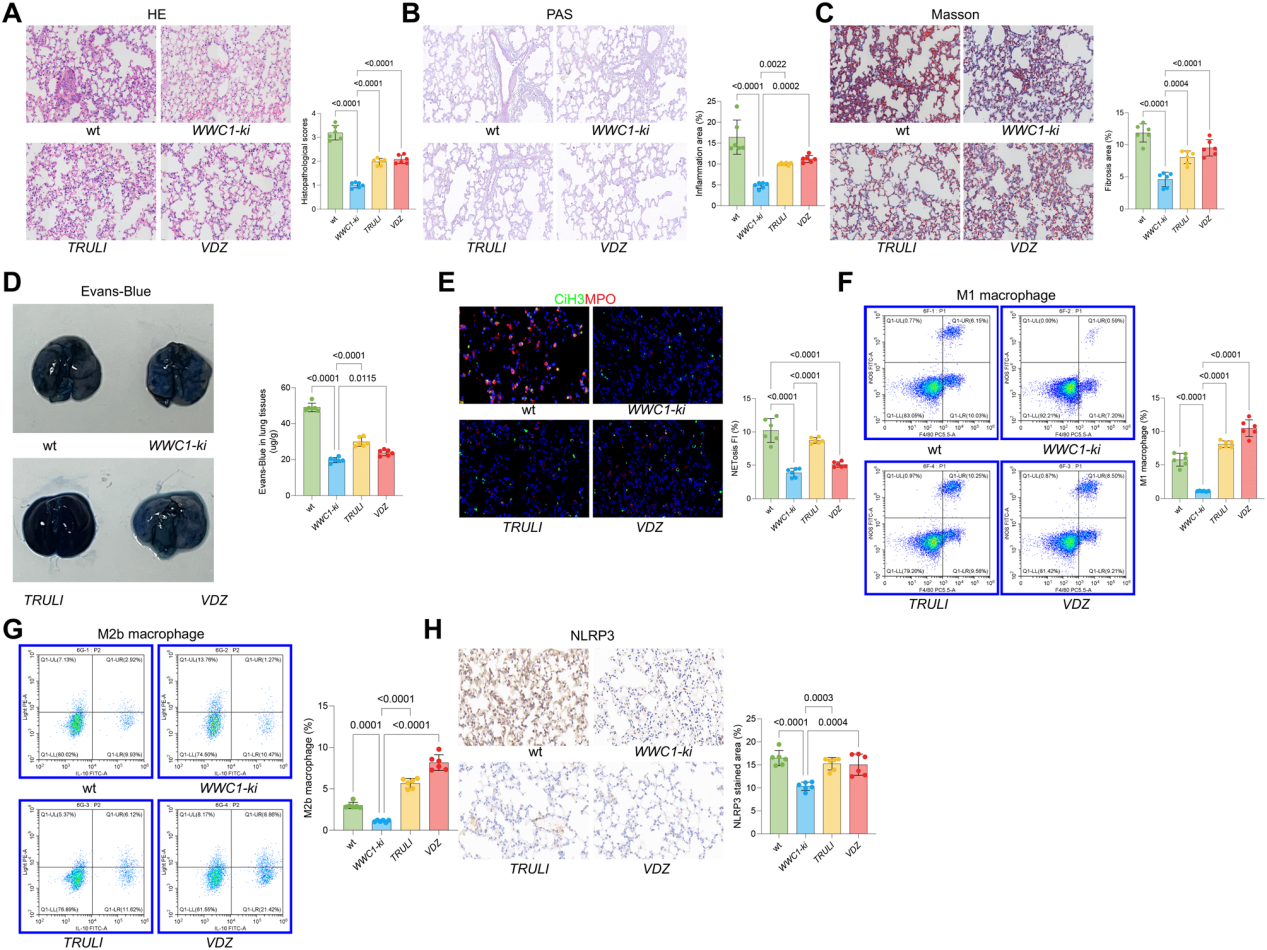
WWC1 regulates pyroptosis and inflammatory cell infiltration via YAP1 and STING pathways. SiLI was generated in wt or WWC1 knock-in (ki) mice through CLP, followed by the treatment of corn oil, the LATS-specific inhibitor TRULI, or the STING agonist VDZ. **A**, HE staining to assess pathological damage in the lung tissue of mice; **B**, PAS staining to evaluate inflammation intensity in the lung tissue of mice; **C**, Masson’s trichrome staining to assess fibrosis in the lung tissue of mice; **D**, Evans blue staining to detect lung tissue permeability in mice; **E**, immunofluorescence analysis of MPO and CiH3 fluorescence intensity in the lung tissue of mice; **F-G**, flow cytometric analysis of M1 (F4/80^+^iNOS^+^) and M2b (CD86^+^IL10^+^Light^+^) macrophages in the BALF of mice; **H**, IHC analysis of NLRP3 expression in lung tissue of mice. Each group contained 6 mice. *p*-values are indicated in the statistical graphs, with *p* < 0.05 considered significant

Compared to wt mice, WWC1-ki mice showed markedly alleviated lung injury, as manifested by reduced inflammation and collagen deposition in the lung tissues. However, further activation of YAP1 or STING resulted in a significant aggravation of tissue damage [Fig. 6A–C]. The WWC1 ki mice also exhibited reduced lung permeability. However, after treatment with TRULI or VDZ, the lung tissue permeability, as indicated by the increased Evans-Blue content, was promoted [Fig. 6D].

The assessment of NETosis showed that the WWC1-ki mice showed significantly reduced immunofluorescence staining intensities of CiH3 and MPO in their lung tissue compared to wt mice. (Fig. 6E). Additionally, the proportions of M1 and M2b macrophages in the BALF of mice were significantly reduced upon WWC1 knock-in [Fig. 6F–G]. However, these trends were reversed by the treatment of either TRULI or STING [Fig. 6E–G]. The YAP1 or STING activation also rescued NLRP3 levels in the lung, which were initially suppressed in the WWC1-ki mice [Fig. 6H].

**Fig. 7:**
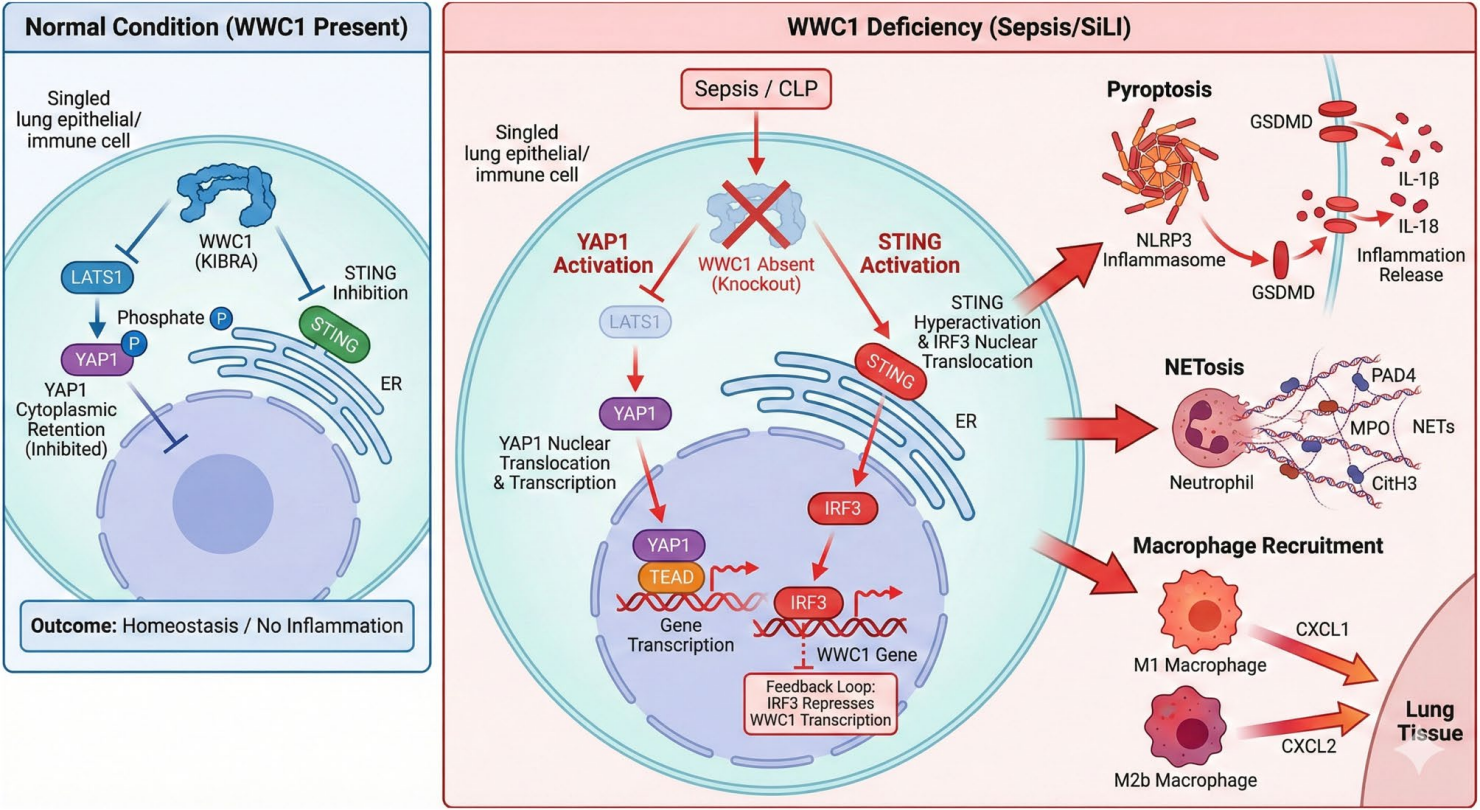
Schematic model of WWC1-mediated regulation in sepsis-induced lung injury (SiLI). Under normal conditions, WWC1 acts as a scaffold to promote LATS1 activation, which phosphorylates and inhibits YAP1. WWC1 also suppresses the cGAS-STING pathway. Upon sepsis challenge (CLP), WWC1 deficiency leads to: 1)Reduced LATS1 activity allows YAP1 to translocate to the nucleus. 2) loss of inhibition leads to STING-IRF3 signaling. These two axes parallelly drive pyroptosis (NLRP3/GSDMD), NETosis (PAD4/CiH3), and the recruitment of M1/M2b macrophages, ultimately exacerbating lung injury

### Combined inhibition of YAP1 and STING exerts additive protective effects

To decipher whether the YAP1 and STING pathways operate independently or synergistically downstream of WWC1 deficiency, we treated WWC1-ko mice with a combination of the YAP1 antagonist CA3 and the STING antagonist C-170. Interestingly, the combined treatment resulted in a more pronounced alleviation of lung tissue damage and a further reduction in the pathological score compared to mice treated with either inhibitor alone [Fig. S3A–B]. Consistent with this, the combined inhibition more effectively suppressed the recruitment of neutrophils and M1 macrophages in the BALF [Fig. S3C–D] and further decreased the levels of NETosis markers (CiH3, MPO) in the lung tissue [Fig. S3E]. These data suggest that WWC1 loss triggers inflammatory cascades through the parallel and additive activation of both YAP1 and STING pathways.

## Discussion

Despite extensive research on the pathological mechanisms of SiLI over the past few decades, a comprehensive understanding of this complex condition remains elusive due to its multifaceted nature and the involvement of numerous cell types [2]. In this study, we present evidence that the activation of the YAP1 and STING pathways, following the loss of WWC1, fulfills a significant function in promoting pyroptosis, NETosis, and the accumulation of neutrophils and macrophages in SiLI.

We first detected increased populations of neutrophils and macrophages in the BALF of WWC1-ko mice compared to wt mice. The activation and migration of neutrophils are key events in the development of ALI [30]. Previous clinical studies have underscored an association between neutrophil levels in the BALF and the severity of disease as well as prognosis in patients with ARDS [31–33]. In animal models, depleting neutrophils has been observed to ameliorate lung injury [34]. Furthermore, blocking interleukin-8 (IL-8), a key chemoattractant for neutrophils, has been protective in rabbits subjected to acid aspiration-induced lung injury [35]. These findings highlight the pathological role of neutrophil infiltration in ALI, a process that also holds true in SiLI, where NETosis exacerbates septic injury by promoting inflammatory cytokines such as IL-1β, IL-8, and tumor necrosis factor-alpha (TNFα) [36]. In addition, we observed elevated expression of MPO, PAD4, and CiH3 in the lung of WWC1-ko mice, as well as increased concentrations of dsDNA and CiH3 in their BALF. These findings strongly support that the loss of WWC1 contributes to neutrophil infiltration and NETosis during the progression of SiLI.

Macrophages are another key group of immune cells responsible for the regulation of inflammatory responses in SiLI [37]. Previous studies have demonstrated a substantial increase in the abundance of total macrophages, particularly the M1 subtype, following exposure to lipopolysaccharides (LPS) [38, 39]. In our analysis of macrophages in the BALF, we observed increase populations in both M1 and M2 macrophages in WWC1-ko mice. However, this classification of macrophages into M1 and M2 phenotypes may be overly simplistic, as these phenotypes represent opposite ends of a continuum of macrophage activation states that occur during homeostasis and inflammation [40]. In fact, macrophages often express genes that blur the line between M1 and M2 subtypes [41, 42]. In our study, further phenotypic analysis revealed an increase in both M1 and M2b macrophages. While M2 macrophages generally promote inflammation resolution and tissue repair, M2b macrophages, which can be induced by immune complexes along with IL-1β or LPS, are more variable in their functions. These cells may exert either anti-inflammatory effects (e.g., IL-10 and IL-12) or pro-inflammatory effects (e.g., TNFα, IL-6, and IL-1β) depending on the cytokines they secrete [43, 44]. In the context of SiLI, the recruitment of both M1 and M2b macrophages likely contributes to the deterioration of lung injury in the absence of WWC1. This was further supported by our findings, which showed that selective inhibition of NETosis or M1 macrophage activation improved the pathological alterations observed in the lungs of WWC1-ko mice.

Our experiments also revealed the significant role of pyroptosis in these processes. The pyroptosis mechanism involves the activation of inflammasomes, particularly the NLRP3 inflammasome [45]. During pyroptosis, caspase-1 is activated, leading to the cleavage and maturation of pro-inflammatory cytokines like IL-1β and IL-18 [46]. Caspase-1 also cleaves GSDMD, generating its N-terminal fragments, which move to the plasma membrane, triggering pyroptosis [47, 48]. We observed the upregulation of GSDMD-N, pro-caspase-1, and NLRP3 in the lungs of WWC1-ko mice, indicating the activation of the pyroptosis pathway upon WWC1 deficiency. Notably, pyroptosis is closely linked to the activity of both neutrophils and macrophages. Pyroptosis in neutrophils has been shown to regulate the production of NETs and is crucial for the onset of sepsis-associated organ impairment [26]. For instance, Xie et al. demonstrated that deleting GSDMD in neutrophils protected against LPS-induced lung injury [49]. Suppressing pyroptosis also reduced the abundance of M1 macrophages in mice with LPS-induced septic lung injury [3]. In line with these observations, we found that treatment with the NLRP3 inhibitor D359-0396 reduced pyroptosis, suppressed NETosis, and decreased macrophage infiltration, leading to alleviation of lung injury in WWC1-ko mice.

The functions of WWC1-associated molecules in pyroptosis, NETosis, and immune cell recruitment were further investigated in our study. We found that the administration of specific antagonists targeting YAP1, STING, and IRF3 significantly reduced pyroptosis, alleviated NETosis, decreased macrophage infiltration, and mitigated lung inflammatory damage in WWC1-ko mice. These findings align with previous studies demonstrating the roles of YAP1, STING, and IRF3 in promoting pyroptosis and inflammatory pathways in various models [23, 50–56]. To further validate these observations, we examined WWC1-ki mice, which exhibited greater resistance to pyroptosis, NETosis, and macrophage recruitment following CLP challenge. However, upon artificial activation of YAP1 or STING, we observed a reactivation of these inflammatory cascades, leading to a return of the previously observed pathological processes. These results suggest that the WWC1-associated molecules exert a crucial function in modulating the inflammatory cascades and that their modulation can significantly influence the severity of pyroptosis, NETosis, and macrophage infiltration in the context of SiLI.

Mechanistically, WWC1 (also known as KIBRA) serves as a critical upstream regulator of the Hippo pathway. It functions as a molecular scaffold that recruits LATS1/2 and MST1/2, facilitating the phosphorylation and activation of LATS1/2. Activated LATS1/2 then phosphorylates YAP1, sequestering it in the cytoplasm and preventing its transcriptional activity in the nucleus. Our data suggest that in the context of SiLI, the loss of WWC1 disrupts this scaffold, leading to reduced LATS1 phosphorylation and the subsequent nuclear accumulation of YAP1. Simultaneously, our previous work has identified a negative feedback loop involving the STING pathway, where the transcription factor IRF3 binds directly to the WWC1 promoter to repress its expression. In the absence of WWC1, this restraint on the cGAS-STING axis is lost, leading to hyper-activation of STING-dependent inflammatory signaling. Therefore, WWC1 acts as a central “brake” on both the YAP1-mediated transcriptional program and the STING-mediated innate immune response.

Our findings hold significant translational promise for the management of sepsis-induced lung injury (SiLI), a condition with limited therapeutic options. Currently, specific inhibitors targeting the YAP1 pathway (e.g., Verteporfin, CA3) and the STING pathway are under active investigation in various clinical and preclinical contexts, primarily for cancer and autoimmune diseases. Our study suggests that these agents could be repurposed to mitigate the excessive inflammation observed in SiLI. Furthermore, the identification of WWC1 as a master regulator suggests that stratifying sepsis patients based on WWC1 expression levels or downstream biomarkers (such as plasma CitH3 or BALF cytokines) could help identify those most likely to benefit from YAP1- or STING-targeted therapies, paving the way for precision medicine in critical care.

In conclusion, this investigation offers new insights into the functions of YAP1 and the STING pathway, activated upon WWC1 loss, in driving inflammatory responses in SiLI, particularly through the promotion of pyroptosis, NETosis, and the pro-inflammatory activation of macrophages [Fig. 7]. Our findings deepen the understanding of how these molecules contribute to the pathological mechanisms of SiLI, highlighting their potential as promising therapeutic targets for managing this condition.

## Electronic supplementary material

Below is the link to the electronic supplementary material.

Supplementary material 1

## Data Availability

The raw data supporting the conclusions of this article are available from the corresponding author upon reasonable request.
