# Supplementary material for: WWC1 deficiency exacerbates sepsis-induced lung injury by promoting NETosis, M1 and M2b macrophage recruitment, and pyroptosis via YAP1 and STING pathways
Source: J Inflamm (Lond). 2026 Apr 17;23:19. doi: 10.1186/s12950-026-00488-8 (PMC13227824; doi:10.1186/s12950-026-00488-8)
Supplement: Supplementary file 1 — Supplementary material 1 [file 12950_2026_488_MOESM2_ESM.docx]

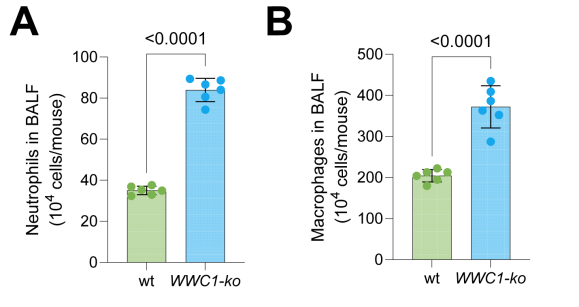


**Fig. S1**. WWC1 knockout promotes recruitment of neutrophils and macrophages in septic mice. SiLI was induced in wt or WWC1-ko mice via CLP. A-B, flow cytometric analysis of the number of neutrophils (Ly6G^+^) and macrophages (F4/80^+^CD11b^+^) in the BALF of wt or WWC1-ko mice. Each group contained 6 mice. *P*-values are indicated in the statistical graphs, with *P* < 0.05 considered significant.


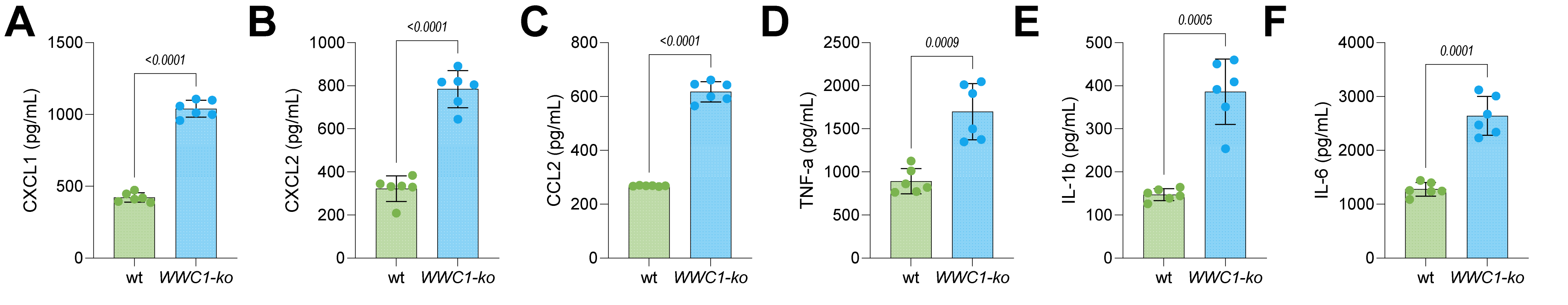


**Fig. S2.** WWC1 knockout elevates pro-inflammatory cytokine and chemokine levels in the BALF. SiLI was induced in wt and WWC1-ko mice via CLP. BALF was collected 24 hours post-operation. A–C, ELISA analysis of chemokines CXCL1 (A), CXCL2 (B), and CCL2 (C) in the BALF. D-F, ELISA analysis of pro-inflammatory cytokines TNF-α (D), IL-1β (E), and IL-6 (F) in the BALF. Each group contained 6 mice. P-values are indicated in the statistical graphs, with P < 0.05 considered significant.


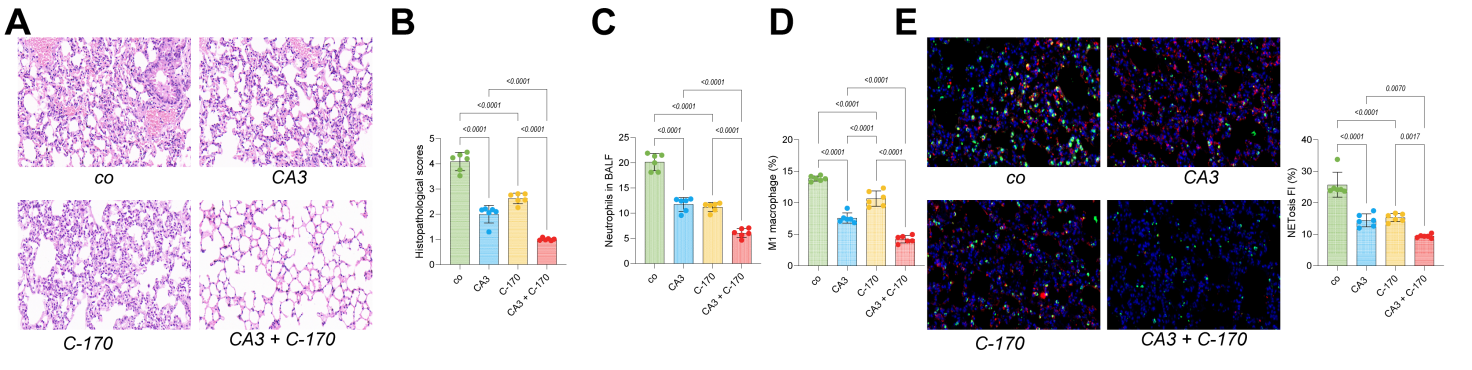


**Fig. S3.** Co-inhibition of YAP1 and STING provides additive protection against SiLI in WWC1-ko mice. WWC1-ko mice were subjected to CLP and treated with CA3 (YAP1 inhibitor), C-170 (STING inhibitor), or a combination of both (CA3 + C-170). A, HE staining of lung tissues. B, Histopathological scoring of lung injury. C-D, Flow cytometric quantification of neutrophils (C) and M1 macrophages (D) in the BALF. E, Immunofluorescence analysis of NETosis markers (CiH3 and MPO) in lung tissue. P-values are indicated in the statistical graphs, with P < 0.05 considered significant.


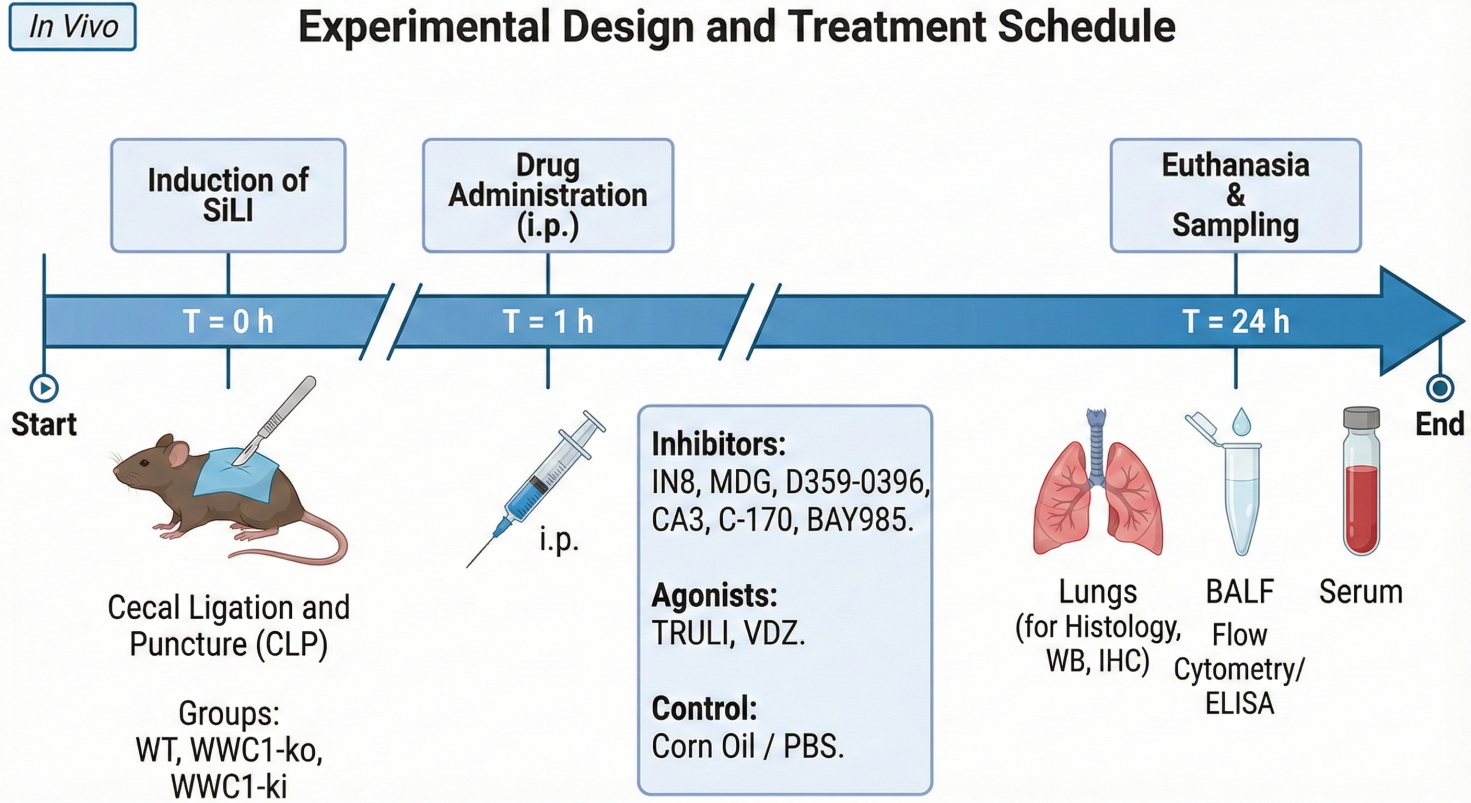


**Fig. S4.** Experimental design and timeline.
